# Assessing Formability and Failure of UHMWPE Sheets through SPIF: A Case Study in Medical Applications

**DOI:** 10.3390/polym15173560

**Published:** 2023-08-27

**Authors:** Ana Rosa-Sainz, M. Beatriz Silva, Ana M. Beltrán, Gabriel Centeno, Carpóforo Vallellano

**Affiliations:** 1 Departamento de Ingeniería Mecánica y Fabricación, Escuela Técnica Superior de Ingeniería, Universidad de Sevilla, 41092 Sevilla, Spain; gaceba@us.es (G.C.); carpofor@us.es (C.V.); 2 Departamento de Ingeniería y Ciencia de los Materiales y del Transporte, Escuela Politécnica Superior, Universidad de Sevilla, 41011 Sevilla, Spain; abeltran3@us.es; 3 Instituto de Engenharia Mecânica (IDMEC), Instituto Superior Técnico, University of Lisbon, Av. Rovisco Pais, 1049-001 Lisboa, Portugal; beatriz.silva@tecnico.ulisboa.pt

**Keywords:** SPIF, UHMWPE, formability, failure, twisting, springback

## Abstract

This work presents a comprehensive investigation of an experimental study conducted on ultra-high molecular weight polyethylene (UHMWPE) sheets using single point incremental forming (SPIF). The analysis is performed within a previously established research framework to evaluate formability and failure characteristics, including necking and fracture, in both conventional Nakajima tests and incremental sheet forming specimens. The experimental design of the SPIF tests incorporates process parameters such as spindle speed and step down to assess their impact on the formability of the material and the corresponding failure modes. The results indicate that a higher step down value has a positive effect on formability in the SPIF context. The study has identified the tool trajectory in SPIF as the primary influencing factor in the twisting failure mode. Implementing a bidirectional tool trajectory effectively reduced instances of twisting. Additionally, this work explores a medical case study that examines the manufacturing of a polyethylene liner device for a total hip replacement. This investigation critically analyses the manufacturing of plastic liner using SPIF, focusing on its formability and the elastic recovery exhibited by the material.

## 1. Introduction

In recent decades, the paradigm of Industry 4.0, characterized by an orientation towards flexible, low to medium volume production processes with a strong emphasis on product customization [1], has instigated profound transformations in manufacturing processes. One representation of this progressive trend can be recognized in the manufacturing process known as Incremental Sheet Forming (ISF), more specifically, in its simpler, dieless variant termed as Single Point Incremental Forming (SPIF). SPIF is a flexible process that relies on simple tool geometries, making it cost-effective. By eliminating the need for a die, significant reductions in tool costs are achieved. In addition, the same setup can be used to produce various geometries, allowing easy customization at minimal costs.

During the last decades, the use of SPIF in the context of polymeric materials has seen a significant increase. This trend can be attributed not only to the aforementioned benefits of the process, but also to experimental validations that demonstrate the strains accomplished by SPIF exceed the Forming Limit Curve (FLC) without any material failure [2,3,4]. As described in Marques et al. [2] for polymeric sheets, the occurrence of necking is often postponed or even suppressed. Consequently, traditional Forming Limits Curve (FLC) is not applicable in this context. Instead, Fracture Forming Line (FFL) is commonly used to determine the formability limits of polymeric sheets within the principal strain space, considering this unique behavior.

In this context, Martins et al. [5] and Marques et al. [2] proposed a methodology to determine the FLC and FFL in thermoplastic materials based on the Circle Grid Analysis (CGA) method. However, it is important to note that the CGA method only provides a static analysis at the end of the test, which limits its ability to accurately track the propagation of necking during typical polymer deformation. Due to the lack of systematic procedures for determining the FLC and FFL in polymeric materials, the authors’ previous work established a comprehensive methodology by adapting techniques used in sheet metal forming to polymer sheets. These methodologies were employed to determine the Forming Limit Diagram (FLD) in 1 mm [6] and 2 mm [7] thickness polycarbonate (PC) sheets, as well as in 2 mm thickness polyether ether ketone (PEEK) sheets [8].

Martins et al. [5] identified the main principal modes of failure in the SPIF process of polymers for conical geometry. The first mode, known as circumferential crack, involves cracks opening along the circumferential direction. The second mode, known as twisting or wrinkling, occurs when the material undergoes twisting within the cone, leading to the formation of wrinkles on the wall. Lastly, the third mode, termed oblique cracking, is characterized by cracks opening along the bisector direction in the inclined wall of the part. Typically, the most common modes of failure observed in SPIF are circumferential cracking and twisting. Le et al. [9] observed both of these failure modes in a cone-shaped part with a circular arc generatrix using a 3 mm thickness polypropylene (PP) sheet. Similarly, Davarpanah et al. [10] conducted tests on two different SPIF geometries, namely variable wall angle and fixed angle conical geometries, using a 0.7 mm thickness polylactic acid (PLA) sheet. In both geometries, circumferential cracking and twisting failures were identified. In addition to the aforementioned failure modes, Rosa-Sainz et al. [7] observed a different failure mode for PC sheets in SPIF known as crazing. The study conducted experiments using PC sheets with a thickness of 2 mm and a truncated conical geometry, varying the spindle speeds at 20, 500, and 1000 rpm. The findings showed that the crazing failure mode specifically occurred at a spindle speed of 1000 rpm. This led to the development of multiple micro-cracks on the deformed specimen. These modes of failure highlight the potential challenges and failure mechanisms associated with the SPIF process in polymer sheets.

As a result of the growth in the development of SPIF process analysis, several research studies have focused on medical applications involving the manufacturing of customized prostheses using incremental forming techniques with polymeric biomaterials. These biomaterials must possess biocompatibility; that is, they should be able to interact long-term with human body tissues without causing any harm [11]. For example, Bagudanch et al. [12], Centeno et al. [13], Clavijo-Chaparro et al. [14], and Chen et al. [15] have conducted research on the implementation of SPIF using polymeric sheets for cranial prostheses in the medical field. Fiorentino et al. [16] present another noteworthy study focusing on the production of biocompatible biomedical devices using ISF with biocompatible polymers. Their research specifically explores the ISF manufacturing process of a palate prosthesis using a combination of titanium alloy and PCL (polycaprolactone), yielding promising results.

In the medical field, the biocompatible polymer Ultra-High Molecular Weight Polyethylene (UHMWPE) is used in various orthopedic applications such as hips, knees, shoulders, elbows, wrists, ankles, and spinal disks due to its exceptional properties [17]. UHMWPE is known for its excellent wear resistance, low friction coefficient, biocompatibility, and high impact strength [18]. These properties make it an ideal material for joint replacement implants and orthopedic devices. Charnley [19] introduced UHMWPE as a bearing component for total joint replacements in 1962, and since then it has become the gold standard for manufacturing articulating surfaces in total hip, total knee, and total shoulder prostheses.

Advancements in polymeric materials led to the use of high-density polyethylene (HDPE) in the 1970s, which exhibited reduced wear rates (around 0.10 mm/year) [20]. In the early 2000s, UHMWPE emerged as a significant development, demonstrating even lower wear rates (<0.02 mm/year) [21]. UHMWPE excels in wear resistance, toughness, durability, and biocompatibility, making it a preferred choice as a bearing material in combination with ceramic or metallic counter surfaces in joint arthroplasty [22]. It is worth noting that over 90% of hip implants in the past decade have utilized UHMWPE [21,23].

Within this context, the present work encompasses three distinct objectives. Firstly, it aims to obtain the forming limit diagram of UHMWPE by employing established methodologies for determining the limits of forming through necking and fracture, as documented in the authors’ previous research [24]. Secondly, to provide a comprehensive investigation on this material behavior subjected to single-point incremental forming. The purpose of this investigation is to assess the process parameters that impact the material in terms of its formability and failure modes. Lastly, to incorporate a practical case within the domain of medical prosthesis manufacturing. Specifically, it focuses on the manufacture of a plastic liner utilized in total hip replacement procedures using the SPIF process with UHMWPE.

## 2. Methods

This section focuses on the methodology employed and is categorized into the following subsections: (i) determination of forming limits, (ii) incremental sheet forming tests, and (iii) medical application of the plastic liner with UHMWPE.

### 2.1. Formability Limits

The ultra-high molecular weight polyethylene (UHMWPE) sheets utilized in this research were procured as 2 mm thickness sheets measuring 500 × 500 mm^2^ from an industrial supplier. Based on the manufacturer’s data sheet provided by Goodfellow in Pittsburgh, PA, USA, these sheets have a density of 0.940 g/cm³, a yield stress of 40 MPa, a Rockwell hardness of R70, and demonstrate a 500% elongation at fracture [25].

**Figure 1:**
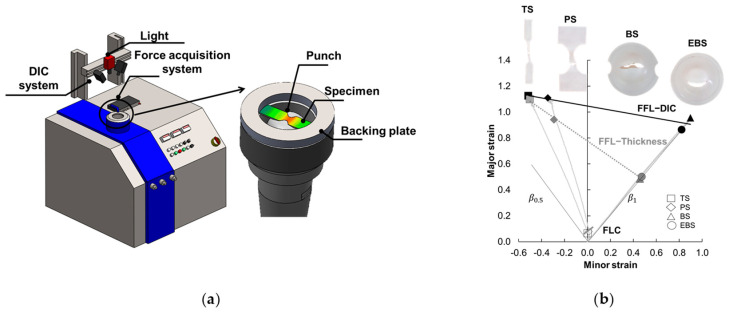
(**a**) Schematic representation of the universal deep drawing machine along with the Nakajima test apparatus. (**b**) Formability limits for 2 mm thickness UHMWPE represented in principal strain space.

To assess the material’s formability limits in terms of necking and fracture, Nakajima tests were conducted. These tests followed the ISO 12004-2 standard [26] and were carried out at room temperature (15–20 °C) using an universal deep drawing machine model 142-20 from ERICHSEN GmbH Co. (Hemer, Germany) (Figure 1a), depicting a tensile strain specimen being deformed by a 100 mm diameter hemispherical punch with a deformation speed of 60 mm/min.

To measure the principal strains within the deforming area near the initial crack zones, an ARAMIS v6.2.0 digital image correlation (DIC) system from GOM (Braunschweig, Germany) was employed. The surface is coated with a black and white stochastic pattern which results in a fine spray of matte black speckles on a white background. The ARAMIS v6.2.0 system consists of two cameras positioned at a relative angle of 23.6° and is equipped with a lens that has a focal length of 50 mm.

The Nakajima specimen geometries were obtained by machining the sheets provided, and these specimens were aligned longitudinally with the extrusion direction. The polymer Nakajima specimens were machined to achieve different strain loading paths from near to tensile strain conditions (approximately $\beta =\frac{\varepsilon_{2}}{\varepsilon_{2}}=-0.3$) towards equibiaxial stretching (β=1). This resulted in various loading paths, including the tensile strain (TS), plane strain (PS), biaxial strain (BS), and equibiaxial strain (EBS).

In this sense, the dimensions of the TS specimen were as follows: the length was 200 mm, the width was 30 mm, the gauge length was 5 mm, the gauge width was 15 mm, and the notch radius was 25 mm. The PS specimen was machined with a length of 200 mm, a width of 114 mm, a gauge length of 4 mm, a gauge width of 45 mm, and a notch radius of 25 mm. As for the BS specimen, it featured a gauge length of 5 mm, a gauge width of 150 mm, a notch radius of 25 mm, and a total diameter of 182 mm. Lastly, the EBS specimen had a total diameter of 182 mm. The dimensions mentioned of the geometries used and the operating conditions can be found in a previous publication by the authors [16]. To minimize friction, two layers of polytetrafluoroethylene (PTFE) were placed between the punch and the polymeric specimen, along with three layers of Vaseline. To obtain statistically meaningful results, each Nakajima test specimen geometry was repeated three times.

The formability limit by necking (FLC) associated with necking was determined in the principal strain space using a time-dependent methodology commonly used for metals [27]. This methodology examines the temporal evolution of the major strain and its first-time derivative (the major strain rate) along a section perpendicular to the crack to identify neck initiation and its progression. On the other hand, the formability limit by fracture (FFL) was determined by measuring the thickness of specimens in the fractured surface using optical microscopy [28]. The minor strain at fracture, $\varepsilon_{2f}^{*}$, and major strain at fracture, $\varepsilon_{1f}^{*}$, were calculated by assuming a constant local loading path slope of the principal strain ratio, $\beta^{*}$, which was determined from the DIC system data ($\beta^{*}=$ ${d\varepsilon }_{2}^{DIC}/d\varepsilon_{1}^{DIC}$). Notably, UHMWPE exhibits significant elastic recovery after fracture due to its relatively low Young’s modulus of elasticity (~1.2 GPa according to the manufacturer). To establish the fracture forming limit line (FFL), the last pair of principal strain values provided by the DIC system before fracture was considered. Further information and details regarding the determination of forming limits for the material can be found in previous works by the authors [6,7].

Figure 1b illustrates the forming limits within the principal strain space for the analyzed 2 mm thickness UHMWPE sheets. The forming limit curve (FLC) in Figure 1b, represented by a grey straight line, was constructed using the strain pairs obtained from the Nakajima tensile strain (TS) and plane strain (PS) Nakajima specimens using the time-dependent approach. The TS strain pairs are depicted as squares, while the PS strain pairs are depicted as rhomboids in Figure 1b. The results obtained from the time-dependent methodology indicated that necking was not the failure mode for the biaxial strain (BS) and equibiaxial strain (EBS) Nakajima specimens.

In terms of the Fracture Forming Limit (FFL) based on thickness, the strain pairs at fracture obtained from the measurement of the fractured specimens were lower than the final points obtained from the Digital Image Correlation (DIC) system. This behavior is similar to what was observed for other materials studied by the authors [6,7,8]. Therefore, it was deemed more appropriate to characterize the FFL for the 2 mm thickness UHMWPE using the FFL−DIC method, represented by a straight line following $\varepsilon_{1}^{DIC}$ = −0.1573$\varepsilon_{2}^{DIC}$+ 1.047.

### 2.2. SPIF Tests

The SPIF experiments were conducted using a spherical end forming tool driven by a CNC milling machine model VMC 100 with 2.5 axes from EMCO GmbH (Hallein, Salzburger) equipped with an EMCOTRONIC TM02 controller. The experiments were performed at room temperature (15–25 °C). The dimensions of the truncated pyramidal geometry are indicated in Table 1. The temperature during the tests was recorded using a FLIR T430sc thermographic camera from Teledyne FLIR LLC (Wilsonville, OR, USA). The thermal camera is attached to a tripod positioned in front of the CNC milling machine, recording the whole surface of the SPIF setup. Temperature data was collected through the following process: multiple points on the surface of the geometries were continuously observed over a period of time. This approach accounted for potential temperature variations caused by factors such as lubricant distribution and tool motion. The selected points were positioned on two separate walls and two distinct corners (specifically for the truncated pyramidal geometry). For each of these areas, the points were positioned at different elevations. The average temperature across all the points was subsequently calculated.

**Figure 2:**
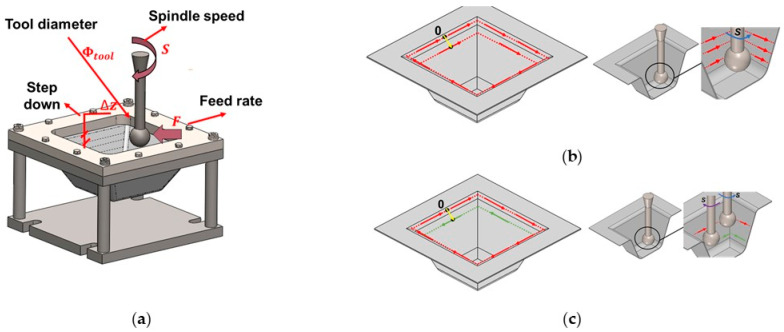
(**a**) Schematic of the SPIF setup and the selected trajectories named: (**b**) unidirectional and (**c**) bidirectional.

Figure 2a illustrates a schematic view of the SPIF setup assembly. The tool followed a predetermined incremental tool path to deform the clamped UHMWPE sheet blank, gradually transforming it into the final truncated pyramid shape. To reduce friction during the forming process, water was applied as a lubricant between the tool and the surface of the sheet. To conduct the SPIF tests, two different toolpaths were utilized: the unidirectional trajectory, denoted as “U”, and the bidirectional trajectory, denoted as “B”. In the case of the unidirectional trajectory, as shown schematically in Figure 2b, the starting point was positioned at the midpoint of the pyramid’s wall instead of a corner to avoid excessive stress concentration at the vertices. The sequence of the unidirectional trajectory was as follows: starting from the designated point, a clockwise trajectory was followed until the starting point again. Then, the tool was incrementally reduced by a certain step down and the clockwise trajectory was repeated. This process was repeated until the final desired shape was achieved. To prevent the occurrence of twisting failure mode [29,30], the tool was rotated in the opposite direction to the trajectory.

The bidirectional trajectory incorporates both clockwise and anticlockwise movements (Figure 2c). The sequence entails initiating a clockwise trajectory from the starting point until returning to the starting point, followed by the descent of the tool. Subsequently, a counter clockwise trajectory is executed. This process is repeated until the desired shape is attained.

The specific operating parameters for UHMWPE used in each experiment are summarized in Table 2. The main focus was to investigate how temperature affects failure modes, particularly twisting. Consequently, the variables analyzed included the influence of the spindle speed and the step down on temperature, as well as the impact of the imposed trajectory on the failure mode in SPIF. To conduct these investigations, the tool diameter was fixed at 10 mm and the feed rate was set at 1000 mm/min. Based on previous experience with polymeric sheet [7,8,31], it was known that spindle speed and step down significantly influence the failure modes. The authors’ previous work [7,31] on PC at 20, 500, and 1000 rpm revealed that very high spindle speeds can lead to a failure mode known as crazing, specifically at 1000 rpm. However, increasing the spindle speed contributed to enhanced formability while reducing geometric precision. In this regard, the intention is to compare two significantly different spindle speed values (20 and 500 rpm) to observe the behavior of UHMWPE in terms of temperature, formability, and elastic recovery.

Additionally, three different step down values were examined: 0.1 mm, 0.2 mm, and 0.5 mm. Two trajectories were studied: the unidirectional trajectory (referred to as “U” in Table 2) and the bidirectional trajectory (referred to as “B” in Table 2). In Table 2, the test condition includes the type of trajectory and the number of the test. To ensure repeatability, three repetitions of each test were performed.

The SPIF tests were conducted until fracture. The resulting outputs considered were the final depth and the specific mode of failure observed during the tests. After the SPIF tests, a qualitative analysis of the specimens’ geometries was performed using a scanner from 3D Systems model Geomagic Capture (Rock Hill, SC, USA).

In addition to the acquired failure modes, a thorough analysis of the strains in SPIF was performed. In previous studies conducted by the authors, a ARGUS system from GOM (Braunschweig, Germany) was used to measure strains after the SPIF deformation process [7,8]. The ARGUS system is an offline 3D deformation digital measurement system that utilizes circle grid analysis (CGA) methodology. To the measurement process, a dot pattern is typically applied to the surface of the specimen prior to the deformation. In the case of UHMWPE, attempts were made to print the dot pattern directly onto the surface of the sheet prior to deformation. However, it was found to be challenging to adhere the dot pattern to the UHMWPE surface during deformation. Various alternatives were explored, including the use of an ink pad and surface fixation treatments, but these attempts proved unsuccessful. The difficulty in applying the dot pattern to UHMWPE is attributed to the high viscosity [32] which restricts its flow and spread, making it unsuitable for conventional ink printing methods. Liquid ink formulations typically require specific viscosity and flow properties to be effectively deposited onto a substrate, but the high viscosity hinders its flowability and prevents successful ink printing.

In this respect, an alternative approach was employed to obtain the strain distribution in the material. This involved measuring the thickness of the SPIFed pyramids after fracture, taking into account the typical modes of deformation observed in a pyramid: plane strain on the walls and biaxial strain at the corners [2]. When the thickness measurements of the fractured pyramids were analyzed, valuable insights could be gained regarding the strain distribution within the UHMWPE material. This approach allowed for an indirect estimation of the strain levels experienced by the material during the SPIF process, particularly in the regions of interest where plane strain and biaxial strain are prevalent, namely the walls and corners of the pyramids, respectively.

### 2.3. Medical Application

**Figure 3:**
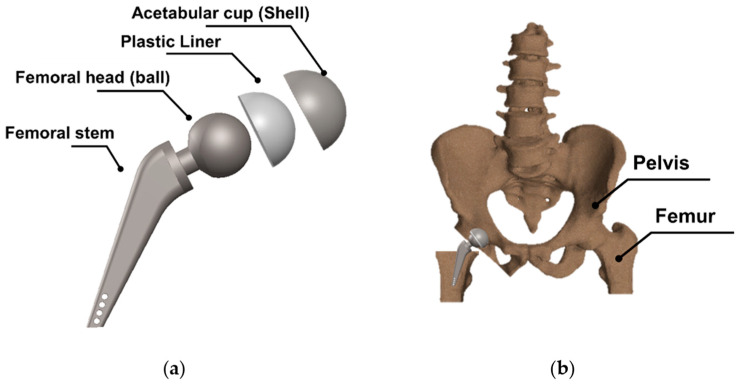
(**a**) Schematic representation of a total hip replacement depicting the different components. (**b**) Assembly of the artificial hip joint prosthesis into the pelvis bone.

Hip replacement surgery is a surgical procedure that is performed to replace deteriorated bone and cartilage within the hip joint with new prosthetic implants. A standard total hip replacement comprises four distinct components, which are shown schematically in Figure 3a: (i) the acetabular cup (shell), which fits in the pelvis and is made of metal; (ii) the acetabular liner, which fits into the acetabular cup; (iii) the femoral head (ball) fits on top of the stem, and the end of the stem is tapered to allow the ball to wedge into position and be held tightly in place with friction; and (iv) the femoral stem is the part of the replacement that fits into the thigh bone [33]. Collectively, these components form a complete hip replacement system (see Figure 3b), reinstating joint functionality and alleviating pain and discomfort associated with hip joint degeneration.

The design of the plastic liner is contingent upon the shell (acetabular cup) used in the hip replacement system. In this regard, various manufacturers provide catalogues specifying the appropriate dimensions for the liner according to the specific shell required for each patient. For the purpose of this study, the Zimmer Biomet Institute (Zählerweg, Switzerland) a manufacturer renowned for its expertise in medical applications, will be used as a reference. The manufacturer provides a catalogue for the acetabular system [34], which includes specific recommendations for the plastic liner. According to the catalogue, a 44 mm plastic liner is recommended for use with a 62 mm shell. The selection of this particular diameter is determined by both the thickness of the plastic sheet and the diameter of the shell. Typically, plastic liners designed for 62 mm shells have a thickness range between 2 and 5 mm. However, it is important to note that larger shell diameters require the use of thicker plastic sheets. This consideration ensures an optimal fit, functionality, and durability of the hip replacement system.

In Table 3, the geometry and dimensions considered for manufacturing the plastic liner device are indicated. In this regard, two different approaches were taken into consideration. According to the catalogue [34], the plastic liners have a geometry comprising a cylindrical wall and a hemispherical zone. This study will begin by examining a purely hemispherical geometry with a 44 mm diameter to observe the behavior of the plastic when subjected to SPIF. Subsequently, the geometry proposed in the catalogue will be considered, which includes a 10 mm cylindrical wall and a hemispherical zone with a 4 mm diameter.

The proposed medical application with UHMWPE considered two premises: (i) minimize the forming time and study the feasibility of manufacturing a plastic liner; and (ii) with the aim of reducing the forming time, improve the springback of the final part produced by SPIF. Therefore, all tests for the case of the hemispherical geometry and the case of cylinder + hemispherical geometry were carried out with a step down of 0.5 mm to reduce the forming time. All tests were performed at a spindle speed of 20 rpm to overheat the material [35,36]. Furthermore, the “cycle” parameter was taken into consideration, which is defined as a complete tool trajectory required to deform the part to its final depth. When the test comprises 3 cycles, it signifies that the forming process is repeated continuously three times. The primary objective of increasing the number of cycles was to enhance the springback characteristics of the material.

All tests were deformed using the bidirectional trajectory according to the results obtained for the pyramidal truncated geometry, and Table 4 indicates the hemispherical geometry (designated as “H” in Table 4) and for cylinder + hemispherical geometry (designated as “CH” in Table 4). To ensure repeatability, two repetitions were performed for each test.

**Figure 4:**
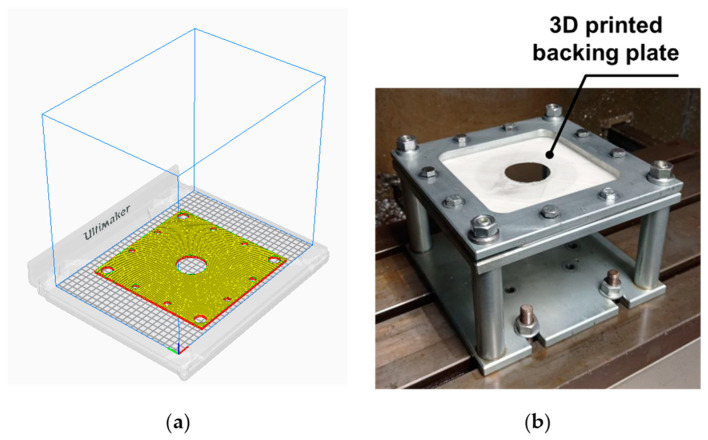
(**a**) 3D model printed using UltiMaker CURA^®^ 4.11 software. (**b**) Mounting of a PLA 3D printed backing plate in the SPIF setup.

A new backing plate was designed and manufactured to better fit the plastic liner device. The design of the backing plate was made using SolidWorks^®^ CAD software 2020 SP 0.0 and fabrication using a 3D printing by Fusion Deposition Modelling (FDM) using PLA filament. The 3D printing process was carried out using a 3D S5 printer from UltiMaker© (Utrecht, The Netherlands), and the UltiMaker CURA^®^ 4.11 software was employed with the following process parameters: the printing temperature is set at 200 °C, whereas the printing speed is 60 mm/s. The chosen pattern for printing is a raster angle of 45°/−45°. The percentage of infill is 100%, indicating that the internal structure of the printed object is completely solid. Figure 4a offers a visualization of the 3D model using UltiMaker CURA^®^ 4.11 software, whereas Figure 4b displays the SPIF setup, focusing on the 5 mm thickness of the 3D printed backing plate.

## 3. Results and Discussion

This section presents the results and discussions obtained, which are divided into two distinct subsections: Section 3.1 focuses on the analysis of formability and failure in the SPIF pyramid tests, while Section 3.2 delves into the results obtained for the manufacturing of the medical prostheses using SPIF.

### 3.1. SPIF Tests

This section provides an overview of the results obtained from the deformation of 2 mm thickness UHMWPE sheets using the SPIF process for the pyramidal geometry. The section is structured into two main parts: (i) an analysis focusing on the formability of the UHMWPE sheets; and (ii) an examination of the temperature data recorded during the SPIF experiments.

#### 3.1.1. SPIF Analysis

The experimental work using the SPIF process is summarized in Table 5. The study involved deforming a truncated pyramidal geometry using two different trajectories: a unidirectional trajectory (trajectory U) and an alternate trajectory (trajectory B) combining clockwise and anticlockwise movements. In this study on UHMWPE, the focus was on investigating the influence of temperature on twisting occurrence and the effect of the trajectory on the observed failure modes. To achieve this objective, a total of nine tests were conducted on UHMWPE sheets, as specified in Table 5.

As explained in Section 2.2, three repetitions of each test were conducted to ensure repeatability. The tests were divided into three categories: three tests following a unidirectional trajectory with a spindle speed of 500 rpm, an additional three tests with a unidirectional trajectory but with a reduced spindle speed of 20 rpm, and finally, three tests performed using a bidirectional trajectory with a spindle speed of 500 rpm. For each test case, the resultant failure mode and the average depth of fracture, derived from the three repetitions, are indicated in Table 5.

**Figure 5:**
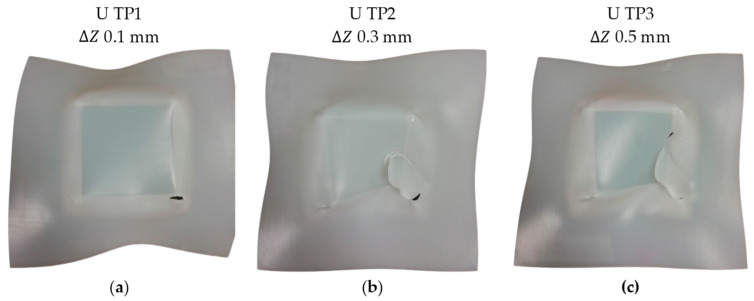
UHMWPE specimens formed by SPIF with the conditions of spindle speed 500 rpm, unidirectional trajectory and step downs of: (**a**) 0.1, (**b**) 0.3, and (**c**) 0.5 mm.

A series of three initial tests was conducted using a unidirectional trajectory (“U”) for the UHMWPE specimens with 500 rpm of spindle speed. The tests were carried out under identical spindle speed, feed rate and tool diameter conditions, with the only variation being the step down values of 0.5 mm, 0.2 mm, and 0.1 mm. Figure 5 illustrates the results obtained from these three specimens. In all three cases, the observed failure modes were a combination of twisting (indicated as “T” in Table 5) and fracture (indicated as “F” in Table 5). It can be observed that the larger step down values corresponded to a higher degree of twisting, as is evident in Figure 5a compared to Figure 5c. The research conducted by Davarpanah et al. 2015 [10] indicated that increasing the step down in SPIF of polymers (PLA and PVC) can have a positive effect in terms of formability. However, it was found that excessively large step down values could cause twisting.

The phenomenon of twisting manifested primarily at the corners of the pyramid due to the inherent stress concentration at these vertices. Moreover, a noticeable trend was observed: decreasing the step-down value resulted in a reduction of the twisting occurrence, as depicted in Figure 5a. In line with this, a recent study by Formisano et al. [37] demonstrated that the twisting originated from the component of forming forces tangential to the toolpath. This specific tangential force led to in-plane shearing, ultimately inducing uncontrolled torsion in the sheets relative to the clamping frame. Additionally, the phenomenon was aggravated by heightened vertical forming forces during polymer sheet shaping. This intensification was attributed to significant indentation, further magnifying the occurrence. In this context, an increase in the step down corresponds to heightened vertical forces [3,10], thereby yielding a more pronounced twisting effect in the UHMWPE specimens.

**Figure 6:**
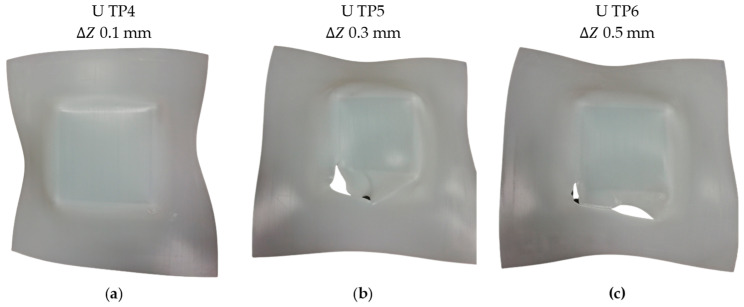
UHMWPE specimens formed by SPIF with the conditions of spindle speed 20 rpm, unidirectional trajectory and step downs of: (**a**) 0.1, (**b**) 0.3, and (**c**) 0.5 mm.

To address the issue of twisting, which is exacerbated by the heating of the contact surface between the tool and the UHMWPE sheet due to friction [9,10], the spindle speed was reduced in an attempt to mitigate this effect. Consequently, another set of three tests was performed using a unidirectional trajectory (“U”) with a spindle speed of 20 rpm. The step down values were varied, ranging from 0.1 mm to 0.5 mm. Similar to the previous tests, the failure mechanism observed in all three cases involved a combination of twisting and fracture, as depicted in Figure 6. It can be observed that the larger step down values resulted in a higher degree of twisting. The twisting phenomenon started at a corner and propagated along the edge, accompanied by the development of cracks. However, it should be noted that the level of twisting observed in these tests was not as prominent as in the cases with a spindle speed of 500 rpm, as shown in Figure 6a–c. In fact, the reduction in spindle speed, along with the smallest step down, led to a significant reduction in twisting in the “U TP4” specimen (Figure 6a), where it is nearly imperceptible.

**Figure 7:**
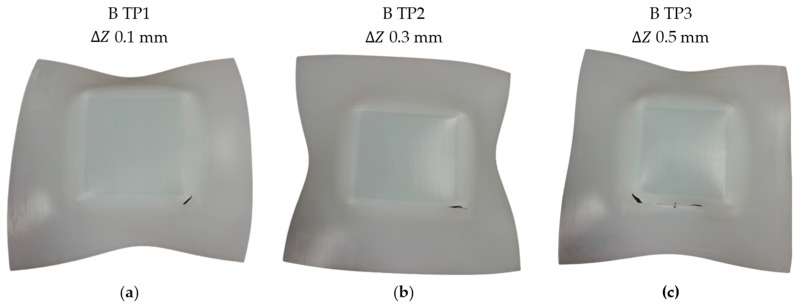
UHMWPE specimens formed by SPIF with the conditions of spindle speed 500 rpm, bidirectional trajectory and step downs of: (**a**) 0.1, (**b**) 0.3, and (**c**) 0.5 mm.

Subsequently, the impact of the trajectory on the failure mode was examined, despite the utilization of a high spindle speed. To investigate this, a series of three tests was conducted using a bidirectional trajectory (“B”) with a spindle speed of 500 rpm. In this regard, the mode of failure associated with the bidirectional trajectory was observed to be solely fracture (Figure 7). The images presented in Figure 7 clearly demonstrate the absence of the twisting mode of failure when using the bidirectional trajectory. In this case, fracture is the sole cause of failure observed. Similar to previous tests, the fracture starts at a corner of the pyramid and proceeds to spread along its edge. In particular, the size of the fracture increases with the larger step down values. In this context, a recent investigation conducted by Yang and Chen [30] unveiled a consistent accumulation of material flow and shear strain aligned with the motion of the tool. Consequently, this phenomenon results in an uneven distribution of thickness within the formed component. In this regard, the inevitable occurrence of twisting is substantiated when utilizing the unidirectional trajectory. Yang and Chen’s investigation involved experimenting with a novel alternative trajectory, employing an interpolation algorithm between three neighboring contour lines. A comparison of their findings demonstrates that this innovative alternate trajectory can significantly enhance the geometric accuracy of the formed parts.

**Figure 8:**
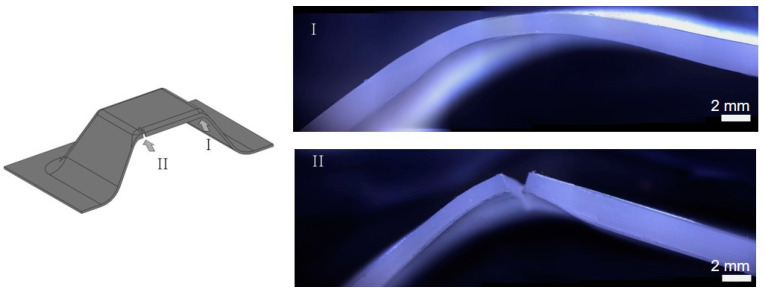
Image of the fracture and non-fracture zones of the UHMWPE SPIFed in the truncated pyramidal geometry. Marker “I” indicates the view of the area without the fracture, whereas “II” points to the section where the fracture is situated.

As explained in Section 2.2, an alternative methodology was followed to obtain the strains for UHMWPE in SPIF. This procedure is based on the work conducted by [5], where measuring the thickness at the fracture is essential for determining the “gauge length” strains. In Figure 8a, a section of the truncated pyramid model is depicted, indicating Zone I (without fracture) and Zone II (with fracture). In Figure 8b, images captured by an optical microscope model SMZ800N from Nikon Corporation (Tokyo, Japan) are presented to provide a clear view of the thickness of the specimen. To accurately measure the thickness, the specimens were cut through a section perpendicular to the crack. The edges of the cut were smoothed using a polisher to ensure precise measurements. Subsequently, the thickness along the section was measured using the microscope. Measurements were taken as close to the fracture region as possible to obtain an accurate representation of the thickness of the fracture. Additionally, various sections without fracture were also measured to verify and corroborate the strain distributions within the material.

**Figure 9:**
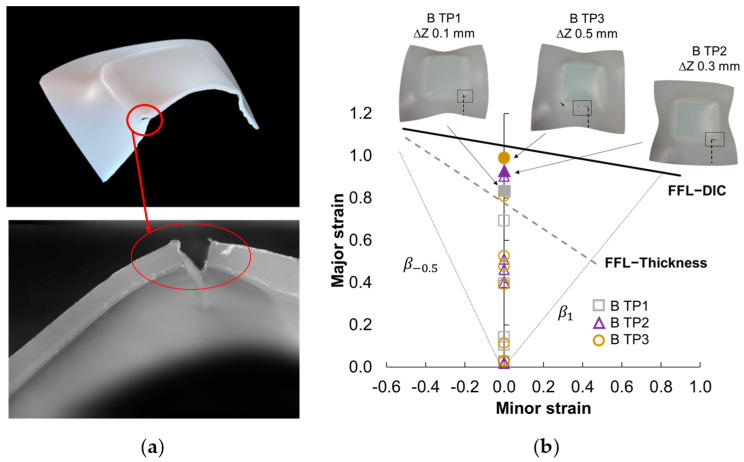
(**a**) Procedure based on the thickness measurement to obtain the strains along a section of the SPIFed specimens. The red circle indicates the fracture that occurred in the biaxial zone (corner) and the plane strain zone (wall). (**b**) FLD for 2 mm thickness UHMWPE sheets within the principal strain space, indicating the three specimens without twisting, highlighting the fracture location, and the section where it was measured using a dashed line.

To assess the magnitude of the major strains in the walls of the truncated pyramidal geometry SPIF tests, a minor strain of zero was assumed to establish a plane strain condition [2]. Additionally, the principle of volume constancy was imposed. As explained previously, the thickness along the fracture section was measured (see Figure 9a for the case of the test B TP1).

Figure 9b illustrates the Forming Limit Diagram (FLD) for the selected SPIF tests without twisting, along with the Fracture Forming Limit (FFL) determined based on the thickness measurements (FFL−thickness) and data from the DIC system (FFL−DIC) obtained under standard Nakajima conditions. The results for the three specific tests, namely “B TP1” with a step down of 0.1 mm (represented by a square marker with grey outline), “B TP2” with a step down of 0.3 mm (represented by a triangle marker with purple outline), and “B TP3” with a step down of 0.5 mm (represented by a circular marker with yellow outline), are presented in Figure 9b. In the FLD, the filled marker represents the strains obtained by measuring the fracture thickness.

As can be observed in Figure 9b, the strains at fracture of the three tests were above the FFL thickness and below the FFL−DIC. In this regard, the FFL−DIC is calculated based on the strains in the last frame measured by the DIC system in such a way that it does not account for the immediate elastic recovery that occurs when the specimen fractures, as it is in the last moment before breaking. On the other hand, the FFL thickness is calculated from the measurement of the thickness of the specimens after the test, such that it does consider this elastic recovery of the material. In the SPIF tests, the thickness along the fracture section has been measured to obtain an order of magnitude of the strains, thus it is measuring while considering the elastic recovery. For that reason, it would be more appropriate to evaluate the strains of the SPIF tests with the obtained FFL thickness, to take into account in both cases the elastic recovery of the material. Consequently, the SPIF fracture strains are above the FFL thickness but below the FFL−DIC, demonstrating an increase in the formability of the material under SPIF.

Furthermore, the comparison between the strains obtained through SPIF and the FFL thickness, based on thickness measurements by Nakajima, reveals that the SPIF test with a step down of 0.5 mm exhibited the highest level of formability. The results of major strain values indicated 0.83 for B TP1, 0.92 for B TP2, and 0.98 for B TP3. An increase in the step down is attributed to the corresponding increase in the maximum attainable wall angle, leading to an improvement in formability. This result is consistent with the research conducted by Bagudanch et al. [3], where an increase in the step down was found to have a positive influence on improving the formability of PVC sheets in SPIF. Similarly, Davarpanah et al. [10] reported similar findings for PLA parts deformed by SPIF, indicating that higher step down values contributed to increased formability.

This investigation provides compelling evidence that the occurrence of twisting, as a mode of failure, can be effectively prevented using the bidirectional trajectory, even with 500 rpm of spindle speed, and even when using a 0.5 mm step down. This significant finding not only enhances the formability of the material but also contributes to reducing the overall duration of the forming process.

#### 3.1.2. Temperature Analysis

This section presents the results of the temperature data analysis during the SPIF tests for the 2 mm thickness UHMWPE truncated pyramidal geometry.

**Figure 10:**
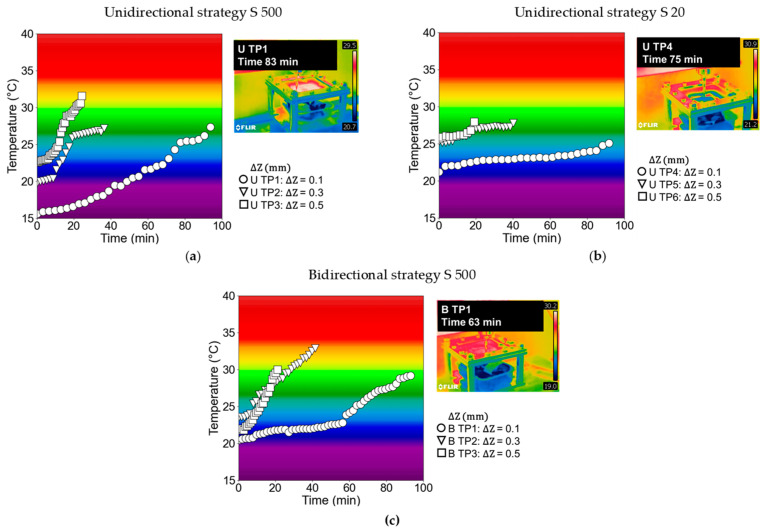
Temperature evolution of UHMWPE for each test in the following scenarios: (**a**) unidirectional strategy with a spindle speed of 500 rpm; (**b**) unidirectional strategy with a spindle speed of 20 rpm; and (**c**) bidirectional strategy with a spindle speed of 500 rpm.

Figure 10 presents the temperature evolution graphs obtained from the truncated pyramidal geometry of SPIF tests conducted on the 2 mm thickness of UHMWPE. The graphs are categorized as follows: Figure 10a represents the unidirectional strategy with a spindle speed of 500 rpm, considering three different step downs (0.1, 0.3, and 0.5 mm); Figure 10b represents the unidirectional strategy with a spindle speed of 20 rpm, also considering three different step downs (0.1, 0.3, and 0.5 mm); and Figure 10c represents the bidirectional strategy with a spindle speed of 500 rpm, again considering three different step downs (0.1, 0.3, and 0.5 mm).

In each graph, the temperature data for the 0.1 mm step down is depicted using circles, the 0.3 mm step down is represented by inverted triangles, and the 0.5 mm step down is represented by squares. Additionally, each graph is accompanied by a temperature contour test image captured using the thermographic camera. These images provide a visual representation of the temperature distribution during the SPIF tests.

In Figure 10a, the temperature data for the unidirectional strategy with a spindle speed of 500 rpm is presented for the tests “U TP1” (0.1 mm step down), “U TP2” (0.3 mm step down), and “U TP3” (0.5 mm step down). It can be observed that “U TP3” (0.5 mm step down) achieved a higher maximum temperature (31.6 °C) compared to the other tests and, correspondingly, it exhibited the shortest forming time (25 min). Conversely, “U TP1” (0.1 mm step down) demonstrated a stable temperature evolution throughout the test, despite using a spindle speed of 500 rpm. Bagudanch et al. [36] observed a similar outcome in their study on UHMWPE, finding that higher values of step down in SPIF led to an increase in temperature levels of the material and a reduced forming time. Similarly, in a recent study conducted by Yang et al. [38] on PEEK, a series of hot SPIF experiments were performed using truncated pyramids with constant wall angles and varying wall angle cones, revealing that as the step down value increased during SPIF for the two geometries, the proportion of the higher temperature range also increased.

Figure 10b illustrates the temperature data obtained using a spindle speed of 20 rpm for the unidirectional strategies of the tests “U TP4” (0.1 mm step down), “U TP5” (0.3 mm step down), and “U TP6” (0.5 mm step down). The temperature evolutions observed in these three tests were similar and demonstrated stability throughout all the tests. Among these tests, “U TP4” (0.1 mm step down) exhibited the lowest temperature, reaching approximately 24 °C. It should be noted that “U TP4” (step down 0.1 and spindle speed 20 rpm) had the least noticeable occurrence of failure mode by twisting on a macroscopic level. This indicates that the combination of a lower spindle speed and a smaller step down value helps to reduce the occurrence of twisting. When a spindle speed of 20 rpm was used, the temperature evolutions for the 0.3 mm and 0.5 mm step downs were similar, resulting in nearly the same maximum temperature levels at the end of the tests, and both presenting twisting as a failure mode. In this sense, as previously mentioned, as the step down value increased, the twisting effect became more pronounced.

As anticipated, the comparison between the results obtained using a spindle speed of 500 rpm and 20 rpm revealed that higher spindle speed values resulted in increased temperature data. This finding aligns with the findings of the study conducted by Lozano-Sánchez et al. [39], which involved the formation of pyramid-shaped parts using UHMWPE through SPIF. Their results demonstrated that an increase in spindle speed led to elevated heat generation due to friction between the tool and sheet. Consequently, in their study, experiments conducted with a spindle speed of 2000 rpm failed due to twisting, unlike those performed with a free rotating tool. Furthermore, Bagudanch et al. [40] also observed that an increase in spindle speed can contribute to a temperature increase during the forming process. This temperature rise is attributed to the intensified friction between the sheet blanks and tools compared to a scenario with a freely rotating tool. Additionally, in the recent work by Formisano et al. [41], the impact of spindle speed on temperature was compared for PC material at 0, 200, and 400 rpm. This study demonstrated that increasing the tool rotation speed can lead to a substantial elevation in the temperature of the contact surface. Furthermore, this study proved that raising the temperature produces both detrimental and beneficial effects. For instance, in the case of PC, processing temperatures higher than 120 °C resulted in severe deterioration. However, it was observed that an increase in temperature can also yield positive outcomes, such as reducing processing loads due to the significant reduction in the flow stress of the polymer, consequently leading to a significant reduction in springback.

On the other hand, Figure 10c provides the temperature data for the bidirectional strategies with a spindle speed of 500 rpm in the tests “B TP1” (0.1 mm step down), “B TP2” (0.3 mm step down), and “B TP3” (0.5 mm step down). Similar to the previous cases, “B TP3” (0.5 mm step down) exhibited a slight increase in temperature data and the shortest forming time compared to the other tests. Tests with 0.1 mm and 0.3 mm step downs displayed comparable temperature evolutions, but in this case, the test with a 0.3 mm step down recorded a higher maximum temperature of approximately 32.9 °C. The experimental studies conducted using the bidirectional approach revealed that fracture was the primary mode of failure, with no evidence of twisting.

Ultimately, a temperature empirical model was developed using Python language [42,43], with the specific purpose of analyzing the variations in temperature within both tool trajectories, taking into account the investigated process parameters. The following equation: $Tmax\left({∆}_{z},S\right)=a\times b^{log\left({∆}_{z}\right)}+c\times S+T_{0}$ reproduces quite well the temperature experimental evolution, where a, b, and c are constants determined to achieve a suitable fit, and T_0_ represents the initial temperature. As observed in Figure 10, the initial temperature ranged from 15 °C to 25 °C, mainly depending on the room temperature within the workspace. An empirical model was developed using various initial temperatures (15, 20, and 25 °C), and the coefficient of determination (R^2^) value approached 1 as the initial temperature increased. The highest R^2^ value achieved was 0.97 when using an initial temperature of 25 °C, indicating that the empirical model provided a closer fit to the experimental results.

Furthermore, a comprehensive examination of the deformed pyramids produced through SPIF is presented, employing a 3D scanner to observe and assess the extent of twisting, whether it demonstrates an increase or decrease. This analysis presents a process window based on the results obtained from deformed pyramids using SPIF with UHMWPE.

**Figure 11:**
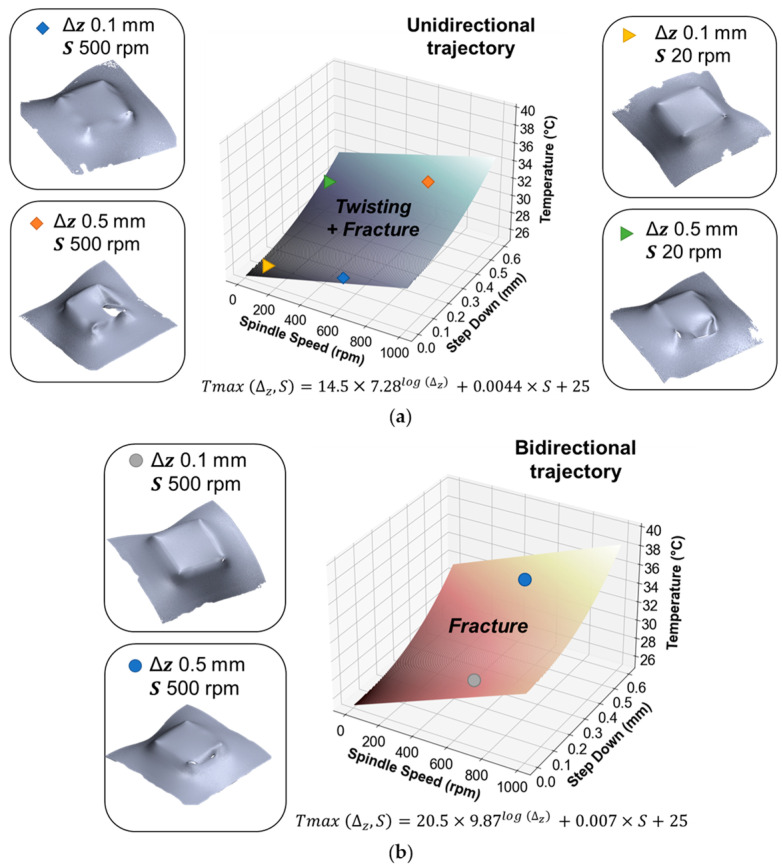
Developed temperature model along with scanned pyramids to illustrate the increase in twisting for the (**a**) unidirectional and (**b**) bidirectional trajectories.

Figure 11a displays the logarithmic surface that fits the temperature results obtained for the unidirectional toolpath. Additionally, the scanned surfaces of the UHMWPE pyramid specimens are shown. It can be observed that for a spindle speed of 20 rpm (triangular markers), an increase in step down leads to an increment in temperature and, in terms of failure modes, a macroscopic increase in twisting. The same trend is observed for a spindle speed of 500 rpm (diamond markers), i.e., an increase in the step down value results in a temperature increment. Figure 11b presents the logarithmic surface obtained for the bidirectional trajectory, as per the detailed equation. Once again, an increase in step down results in elevated temperature levels. However, it was possible to inhibit twisting, leading only to a fracture failure mode.

This analysis highlights that, with equal process parameters and therefore similar temperature levels, utilizing the bidirectional toolpath enables achieving only fracture as the failure mode, even with moderately high spindle speeds.

### 3.2. Plastic Liner Device

This section presents the results of manufacturing a plastic liner device using UHMWPE for joint replacements. As explained in Section 2.3, two different geometries were considered: a hemispherical geometry (“H” in Table 6) and a geometry with a cylindrical and a hemispherical zone (“C + H” in Table 6).

Based on the findings from the UHMWPE truncated pyramidal geometries produced through SPIF, it was determined that the bidirectional trajectory effectively prevents the occurrence of twisting failure, even with higher step down values (0.5 mm). Additionally, the experiments showed that using a lower spindle speed (20 rpm) resulted in a reduction of twisting failure.

With these considerations, the goal of manufacturing the plastic liner device was to establish a foundation for future improvements. Therefore, a step down value of 0.5 mm was chosen to expedite the forming process. The tests were conducted at a spindle speed of 20 rpm to minimize the temperature levels. Furthermore, a three-cycle process was used to achieve greater geometric accuracy and reduce springback, resulting in a shape that closely matches the design specifications in CATIA V5R20.

Table 6 shows the obtained results, indicating that the three plastic devices were successfully manufactured by SPIF without failure.

The alternative approach was employed to assess the formability levels. A meridional section along the specimen was selected after the plastic device and divided into distinct sections where the thickness was measured.

**Figure 12:**
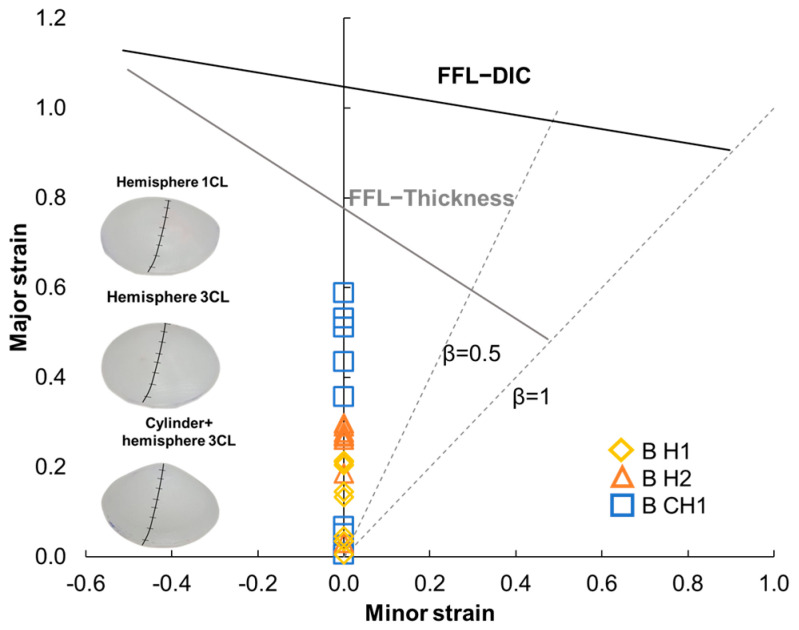
Strains achieved for the UHMWPE SPIF plastic liner devices by using the measurement of the final thickness in the FLD.

In the work by Jeswiet et al. [44], a hemispherical part was manufactured using SPIF. The study revealed that the value of β, representing the principal strain ratio, can range between 0 and 0.1, indicating a near plane strain condition. This information was considered in the analysis of the major strain distribution in the plastic liner device. Figure 12 presents the FLD considering a plane strain condition (β = 0) for the three specimens considered.

The cylindrical plus hemispherical geometry, with three cycles of processing (represented by a square marker with blue outline), exhibited more deformation compared to the hemispherical geometry with 3 cycles (represented by a triangle marker with orange outline), and with one cycle (represented by a rhomboid marker with yellow outline). In terms of strains, B H1 exhibited a value of 0.20, B H2 showed 0.28, and B CH1 displayed 0.58 in the major strain. In all the tests conducted, the maximum strains observed were well below the FFL thickness and/or FFL−DIC, indicating that the manufacturing of the plastic liner device using UHMWPE was feasible.

**Figure 13:**
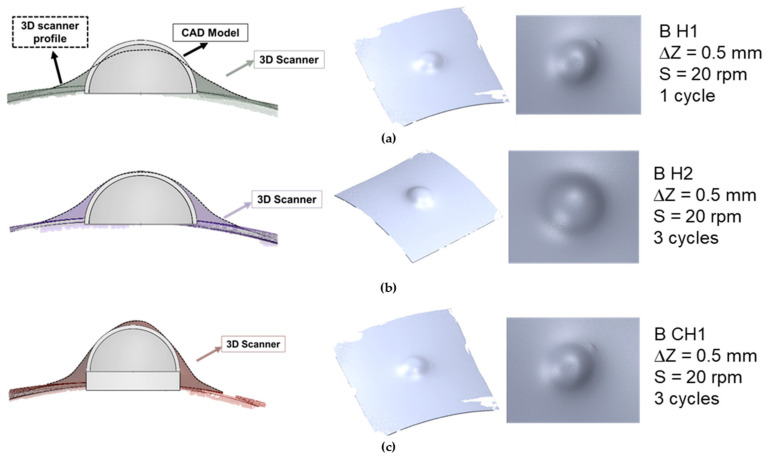
Differences between the 3D scanner plastic liner model and CAD model, accompanied by the image extracted from the 3D Geomatic Capture scanner software, 2016 version for the following cases: (**a**) hemispherical geometry with 1 cycle; (**b**) hemispherical geometry with 3 cycles; and (**c**) hemispherical and cylindrical geometry with 3 cycles.

A qualitative analysis of the plastic liner geometries was performed using a 3D scanner. The process involved overlaying the CAD model with the real model, as depicted in Figure 13.

As anticipated, the three conditions tested for the plastic liner geometries did not result in a perfect fit with the CAD model, primarily due to the material’s elastic recovery. Elastic recovery was most prominent in the curved areas of the plastic liner, particularly along the sides. The hemispherical geometry with only one cycle exhibited the poorest geometric accuracy, while the condition with a combination of the hemispherical and cylindrical wall geometry showed better accuracy. However, it was observed that the top area of the plastic liner was not perfectly hemispherical. This discrepancy may be attributed to the influence of the tool in that particular region.

It has been established that the geometry of the hemisphere plus cylinder with three cycles achieved the highest level of geometric accuracy. However, as discussed previously, the UHMWPE material exhibits significant elastic recovery. To address this issue, further experimentation with alternative geometries and strategies is required. This study is an initial investigation to demonstrate the feasibility of producing UHMWPE-based medical devices using the SPIF process at room temperature. In this scenario, the recent research led by Cheng et al. [45] revealed that the variation in the shape of implants produced using incremental sheet forming could be controlled within the range of 1–2 mm. Nonetheless, additional efforts were required to enhance the geometric accuracy to ensure a properly fitting implant. Furthermore, optimizing the tool path emerged as the most appropriate and efficient strategy for enhancing precision, in contrast to methods such as providing additional support or utilizing hot incremental forming.

## 4. Conclusions

This article examines the formability and failure behavior of UHMWPE polymer through both conventional and incremental single point forming. Material characterization adapts techniques from sheet metal forming, utilizing a successful time dependent approach to detect the onset of necking. The forming limits for necking and fracture in 2 mm thickness UHMWPE sheets were determined. The results indicated that: (i) tensile and plane strain specimens exhibited necking, whereas biaxial and equibiaxial strain specimens did not; and (ii) the latest measurement from the DIC system was utilized to determine the FFL due to the significant elastic recovery presented by the material.

On the other hand, an experimental plan of SPIF was conducted using a truncated pyramid. The research aimed to compare failure modes and formability for step down values of 0.1, 0.3, and 0.5, spindle speeds of 20 and 500 rpm, and unidirectional and bidirectional toolpaths. Under the unidirectional trajectory, the key findings were: (i) twisting and fracture were observed across all conditions; (ii) reducing step down and spindle speed decreased twisting; and (iii) regarding temperature, the highest reached was 31.6 °C in the test with 0.5 mm step down and 500 rpm spindle speed. Under the bidirectional trajectory, the conclusions were as follows: (i) it was possible to avoid twisting, with fractures occurring only under the tested conditions; and (ii) in terms of formability, an increase in step down led to higher levels of formability, with a major strain of 0.98 for the 0.5 mm step down test and 0.83 for the 0.1 mm test (an increase of 15.3%).

The article focuses on the manufacturing of a UHMWPE plastic liner using SPIF, which is a highly utilized medical component in total hip replacement. The results indicated that the part with cylindrical and semi-spherical geometry, subjected to 3 forming cycles, exhibited lower geometric deviations. In terms of strains, the manufactured part reached a major strain level of 0.58, falling below the FFL and demonstrating the potential for manufacturing medical prostheses through incremental forming. Additionally, this article critically considers that the elastic recovery of the material is a potentially improvable parameter for the manufacturing of medical prostheses using UHMWPE.

## Figures and Tables

**Table 1:** Truncated pyramidal geometry dimensions.

Geometry	Dimensions						Wall Angle
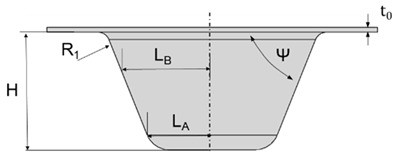	L_A_ (mm)	L_B_ (mm)	R_1_ (mm)	H (mm)	ψ	t_0_ (mm)	Fixed
	25	40	5	50	20	2	

**Table 2:** Experimental SPIF work plan for UHMWPE sheets.

Test Condition	$\Phi_{tool}$ (mm)	S (rpm)	F (mm/min)	z (mm)
U TP1	10	500	1000	0.5
U TP2	10	500	1000	0.3
U TP3	10	500	1000	0.1
U TP4	10	20	1000	0.5
U TP5	10	20	1000	0.3
U TP6	10	20	1000	0.1
B TP1	10	500	1000	0.5
B TP2	10	500	1000	0.3
B TP3	10	500	1000	0.1

**Table 3:** Geometrical parameters for the two considered manufacturing geometries of a plastic liner using UHMWPE.

Geometry			Parameters (mm)		
Hemispherical Geometry			Dimensions		
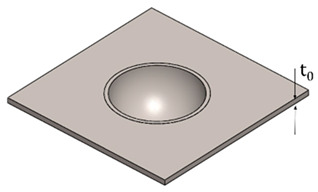	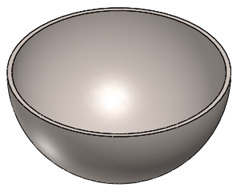	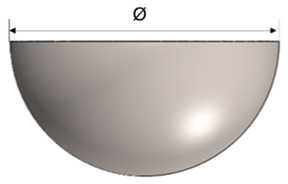		Ø	t_0_
				44	2
Cylindrical and a hemispherical zone			H	Ø	t_0_
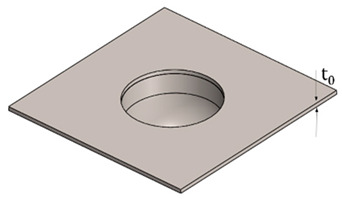	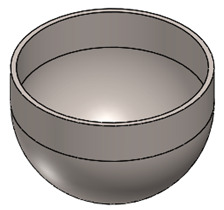	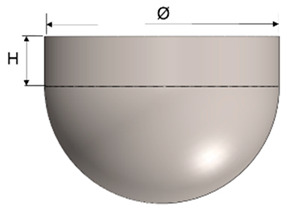	10	44	2

**Table 4:** Process parameters for the two considered manufacturing geometries of a plastic liner using UHMWPE.

Test Condition	$\Phi_{tool}$ (mm)	S (rpm)	F (mm/min)	z (mm)	Cycles
B H1	10	20	1000	0.5	1
B H2	10	20	1000	0.5	3
B CH1	10	20	1000	0.5	3

**Table 5:** SPIF results for the truncated pyramidal geometry with a 2 mm thickness of UHMWPE.

Test Condition	$\Phi_{tool}$ (mm)	S (rpm)	F (mm/min)	∆Z (mm)	Failure	Fracture Depth (mm)
U TP1	10	500	1000	0.1	T + F	29.7
U TP2	10	500	1000	0.3	T + F	36.3
U TP3	10	500	1000	0.5	T + F	39
U TP4	10	20	1000	0.1	T + F	29.4
U TP5	10	20	1000	0.3	T + F	40
U TP6	10	20	1000	0.5	T + F	32
B TP1	10	500	1000	0.1	F	28.1
B TP2	10	500	1000	0.3	F	39
B TP3	10	500	1000	0.5	F	37.5

**Table 6:** Results for hemispherical and hemispherical + cylinder plastic liner with UHMWPE in SPIF.

**Test Condition**	Δz **(mm)**	S **(rpm)**	**Failure**	**Time** **(min)**	**Number of Cycles**
B H1	0.5	20	no	5	1
B H2	0.5	20	no	16	3
B CH1	0.5	20	no	21	3

## Data Availability

The data presented in this study are available on request from the corresponding author.
